# HIV gp120/Tat protein-induced epithelial–mesenchymal transition promotes the progression of cervical lesions

**DOI:** 10.1186/s12981-023-00577-1

**Published:** 2023-11-19

**Authors:** Peizhi Wang, Baojun Yang, Huang Huang, Peiyi Liang, Bin Long, Lin Chen, Lijie Yang, Lianhua Tang, Liping Huang, Huichao Liang

**Affiliations:** 1 Department of Obstetrics and Gynecology, Nanfang Hospital, Southern Medical University, Guangzhou, Guangdong China; 2 Department of Gynecology, Guangzhou Eighth People’s Hospital, Guangzhou Medical University, Guangzhou, 510000 China; 3 Department of Intensive Care Unit, Guangzhou Eighth People’s Hospital, Guangzhou Medical University, Guangzhou, 510000 China

**Keywords:** HIV infection, gp120 protein, Tat protein, Epithelial–mesenchymal transition (EMT), Cervical cancer

## Abstract

**Background:**

Human immunodeficiency virus (HIV) infection is associated with an elevated incidence of cervical cancer, and accelerated disease progression, but the underlying mechanisms are not well understood. This study aimed to investigate the relationship between HIV infection and epithelial–mesenchymal transition (EMT) in cervical cancer.

**Methods:**

Tissue samples from HIV-positive and negative patients with cervical intraepithelial neoplasia (CIN) and cervical cancer were analyzed for EMT-related proteins. Human cervical cancer SiHa cells were treated with HIV Tat and gp120 proteins to test their effects on EMT, migration, and invasion.

**Results:**

HIV-positive patients had lower E-cadherin and cytokeratin, and higher N-cadherin and vimentin levels than HIV-negative patients. HIV Tat and gp120 proteins induced EMT, migration, and invasion in SiHa cells. Transcriptome sequencing analysis revealed that, compared to the control group, the protein-treated group showed upregulation of 22 genes and downregulation of 77 genes. Gene Ontology (GO) and Kyoto Encyclopedia of Genes and Genomes (KEGG) enrichment analyses revealed the involvement of the Wnt signaling pathway in EMT. Further analysis of gene expression related to this pathway revealed upregulation of DVL1, TCF7, KRT17, and VMAC, while GSK3β, SFRP2, and CDH1 were downregulated. Immunofluorescence assay demonstrated that HIVgp120 and Tat proteins treatment induced elevated β-catenin expression with nuclear accumulation in SiHa cells.

**Conclusions:**

The treatment of SiHa cells with HIV Tat and gp120 proteins induces EMT and activates the Wnt/β-catenin pathway, suggesting that the Wnt/β-catenin pathway may play a crucial role in promoting EMT progression in cervical lesion tissues of HIV-infected patients.

## Background

Acquired immunodeficiency syndrome (AIDS)-defining cancers have been demonstrated as a significant contributor to both hospitalization rates and mortality among individuals living with human immunodeficiency virus (HIV) [1, 2]. Cervical cancer is one of the AIDS-related tumors [3], contributing significantly to the burden of cancer among HIV-positive individuals. Stelzle et al. conducted a comprehensive meta-analysis involving 24 studies and 236,127 women with HIV and found that women with HIV had a sixfold increased risk of cervical cancer [4].

In addition, studies have shown that HIV-positive women experience cervical cancer at an earlier age compared to HIV-negative women, with an average age difference of 10 years [5, 6]. Furthermore, HIV infection is associated with a heightened susceptibility to contracting human papillomavirus (HPV) infection, reduced likelihood of pre-cancerous lesions regressing, a more rapid progression toward cervical cancer, and elevated rates of recurrence post-treatment [7, 8], suggesting a potential influence of HIV on the progression of cervical lesions. However, the specific mechanisms that underlie this association are still not well understood.

Despite the recognition of this association between HIV infection and cervical cancer, the underlying mechanisms remain poorly understood. Understanding these mechanisms is crucial for improving prevention and treatment strategies for HIV-positive women. On the other hand, A meta-analysis by Kelly et al. reported that antiretroviral therapy was linked to a reduced prevalence of high-risk HPV and a significantly decreased risk of cervical cancer among women with HIV [9]. These findings indicate that the progression of HIV-related cervical cancer is not solely a result of immune suppression caused by HIV infection.

Epithelial–mesenchymal transition (EMT) is a complex biological process involving the transformation of epithelial cells into mesenchymal cells, characterized by reduced cell adhesion and increased cell motility [10]. EMT has been recognized as a crucial factor in tumor progression, invasion, and metastasis [11, 12]. Understanding the relationship between HIV infection, EMT, and cervical cancer could provide insights into the mechanisms driving the development and progression of cervical lesions in HIV-positive individuals. Therefore, this study aimed to investigate the association between HIV infection and EMT in cervical cancer.

## Materials and methods

### Patients and tissue samples

The cervical specimens, including cases of CIN and cervical cancer, were obtained from patients treated at the Eighth Affiliated Hospital of Guangzhou Medical University in Guangzhou, China, between 2018 and 2022. A total of 118 patients with cervical intraepithelial neoplasia (CIN) grades I–III were included in the study, comprising 54 HIV-positive and 64 HIV-negative patients. Additionally, 48 cervical cancer patients were included, including 26 HIV-infected patients and 22 HIV-negative patients. This study was approved by the Research Ethics Committee of the Eighth Affiliated Hospital of Guangzhou Medical University (Approval No. 202215227). Written informed consent was obtained from patients.

### Immunohistochemistry

The cervical lesion specimens used in this study were surgical resection specimens. Following surgical removal, the specimens were fixed with 10% neutral formaldehyde. Immunohistochemistry was performed on 8 μm serial sections mounted on glass slides using a single-staining procedure. The protocols for each antibody were executed following the instructions provided in the Immunohistochemistry kit (Cat. No. PV-9000, Beijing Zhongshan Jinqiao Biotechnology Co. Ltd [ZSGB-BIO], China). The primary antibodies used were E-Cadherin (ZSGB-BIO), cytokeratin (broad-spectrum, ZSGB-BIO), vimentin (ZSGB-BIO), and N-Cadherin (Zhongshan Aoquan Medical Technology Co., Ltd., China).

Comprehensive analysis and scoring were conducted based on the staining intensity and the number of stained cells. The intensity was assessed using a scoring system with values ranging from negative (0) to strong (3). The proportion of staining was scored as follows: 1 (≤ 10%), 2 (11–50%), 3 (51–75%), and 4 (> 75%). An overall expression score was calculated by multiplying the scores for intensity and proportion, resulting in a range of 0 to 12.

### Cell lines and cell culture

The SiHa cells and human cervical epithelial cells were obtained from Procell Corporation, Wuhan, China. The SiHa cells were cultured in complete growth medium (Procell Corporation) in a humidified incubator at 37 °C with 5% CO_2_.

### Western blot

The protein was extracted using RIPA lysis buffer, and protein concentration was determined using the Bicinchoninic Acid (BCA) Protein Assay Kit (Thermo Fisher Scientific, USA). The protein sample was separated by SDS-PAGE and subsequently transferred onto PVDF membranes (Millipore, MA, USA). To block nonspecific binding, the membranes were incubated with 5% nonfat milk at room temperature for 2 h, followed by washing with Tris-buffered saline-Tween. Primary antibodies against vimentin (1:1000, Signalway Antibody, USA), N-cadherin (1:3000), E-cadherin (1:10,000), cytokeratin (1:2000, HUABIO, Hangzhou, China), and GAPDH (1:1000, Abcam, USA) were incubated with the membranes overnight (16 h) at 4 °C. Subsequently, the membranes were incubated with the appropriate secondary antibody for 2 h at room temperature. Finally, the immunoreactive proteins were visualized using the ECL detection system (Millipore, MA, USA).

### Wound-healing assay

SiHa cells were seeded in a 6-well plate at a density of 1 × 10^5^ cells. The experiment consisted of two groups: a control group and an HIV gp120 and Tat protein treatment group, with each group containing three replicate wells. The treatment group was exposed to 100 ng/ml of HIV gp120 protein and 100 ng/ml of Tat protein (Prospec, USA). The culture medium was replaced daily, and the proteins were added again during each medium change. When the cells reached 90% confluence, wounds were created by scratching a straight line using a 200 µl pipette tip. The wound areas were then imaged at 0 h, 24 h, and 48 h using a microscope. The Image J software (NIH, USA) was utilized to calculate the wound areas.

### Transwell assays

SiHa cells were seeded in a 6-well plate at a cell density of 1 × 10^5^ cells. The experiment consisted of two groups: a control group and an HIV gp120 and Tat protein treatment group, with each group containing three replicate wells. The treatment group was exposed to 100 ng/ml of HIV gp120 protein and 100 ng/ml of Tat protein. The culture medium was changed daily, and the proteins were replenished during each medium change. After 5 days of treatment, cells were collected for the Transwell assay. In the lower part of the Transwell chamber, 700 µl of complete medium was added, while in the upper part, 200 µl of serum-free medium containing suspended cells (1 × 10^4^ cells) was added. The cells were allowed to culture overnight, approximately 18 h, after which, the chamber was removed and fixed with 4% paraformaldehyde for 10 min. Subsequently, the cells were stained with crystal violet for 10 min. Following staining, the cells were rinsed with running water, and a gentle rub with a cotton swab was performed to remove the cells that did not transfer to the small chamber. The cells were then counted under a microscope, with nine randomly selected non-overlapping fields of view used for the analysis.

### RNA sequencing and bioinformatics analysis

SiHa cells were seeded in a 6-well plate at a cell density of 1 × 10^5^ cells. The experiment consisted of two groups: a control group and an HIV gp120 and Tat protein treatment group, with each group containing three replicate wells. The samples from each group were sent to Gene Denovo Biotechnology Co. (Guangzhou, China) for mRNA sequencing analysis. Total RNA from each sample was isolated using the TRIzol reagent kit (Invitrogen, USA). RNA quality was assessed using an Agilent 2100 Bioanalyzer (Agilent. Technologies, Inc., USA), and agar gel electrophoresis was performed to verify RNA integrity. An oligo(dT) bead enrichment procedure was conducted to extract the target mRNA fragments, which were then reverse-transcribed into double-stranded cDNA (ds-cDNA) using random primers. The cDNA fragments were purified, repaired, poly(A)-tailed, and ligated to construct the library. The library was sequenced using the Illumina NovaSeq 6000 platform (Illumina Inc., USA). Differential expression analysis of the RNA-seq data between the two groups was performed using DESeq2 software (University of California, Berkeley, United States). Significantly differentially expressed genes/transcripts were identified based on a false discovery rate (FDR) below 0.05 and an absolute fold change of ≥ 1.5. Gene Ontology (GO) and Kyoto Encyclopedia of Genes and Genomes (KEGG) pathway enrichment analyses were conducted.

### Immunofluorescence

For immunofluorescence labeling and detection, SiHa cells were cultured on coverslips and fixed with 3% formaldehyde in PBS. Permeabilization was achieved by treating the cells with 0.1% saponin. The cells were then blocked with 10% donkey serum at room temperature for 30 min, followed by incubation with anti-β-Catenin antibody (Proteintech, USA; 1:200 dilution) at 4 °C overnight. After washing with PBS 3 times, the cells were incubated with Cy3-conjugated goat anti-rabbit antibodies (Abcam, UK; 1:3000 dilution) at room temperature for 50 min. The cover slips were then mounted using Fluoroshield with DAPI (Sigma, USA). Cell immunofluorescence imaging was observed using a Nikon Eclipse Ti Confocal Laser Scanning Fluorescence Microscope (Nikon, Japan).

### Statistical analysis

Continuous data were indicated with mean ± SD (standard deviation) while categorical data were indicated with number and percentage (%). For comparisons of means between two groups, the student’s independent t-test was used. Categorical data were tested using the Chi-square test or Fisher’s exact test (if an expected value ≤ 5 was found). For comparisons of means among multiple groups equal to or larger than 3, one-way analyses of variance (ANOVA) with Fisher’s Least Significant Difference (LSD) post hoc multiple comparisons was used. A P value < 0.05 was considered statistically significant. All the above analyses were performed using IBM SPSS Version 25 (SPSS Statistics V25, IBM Corporation, Somers, New York). After RNA sequencing, the differential gene expression RNA-seq was analyzed using a difference comparison volcano map, differential gene clustering heat map, and GO enrichment analysis.

## Results

### HIV infection induced EMT in cervical tissue

**Fig. 1:**
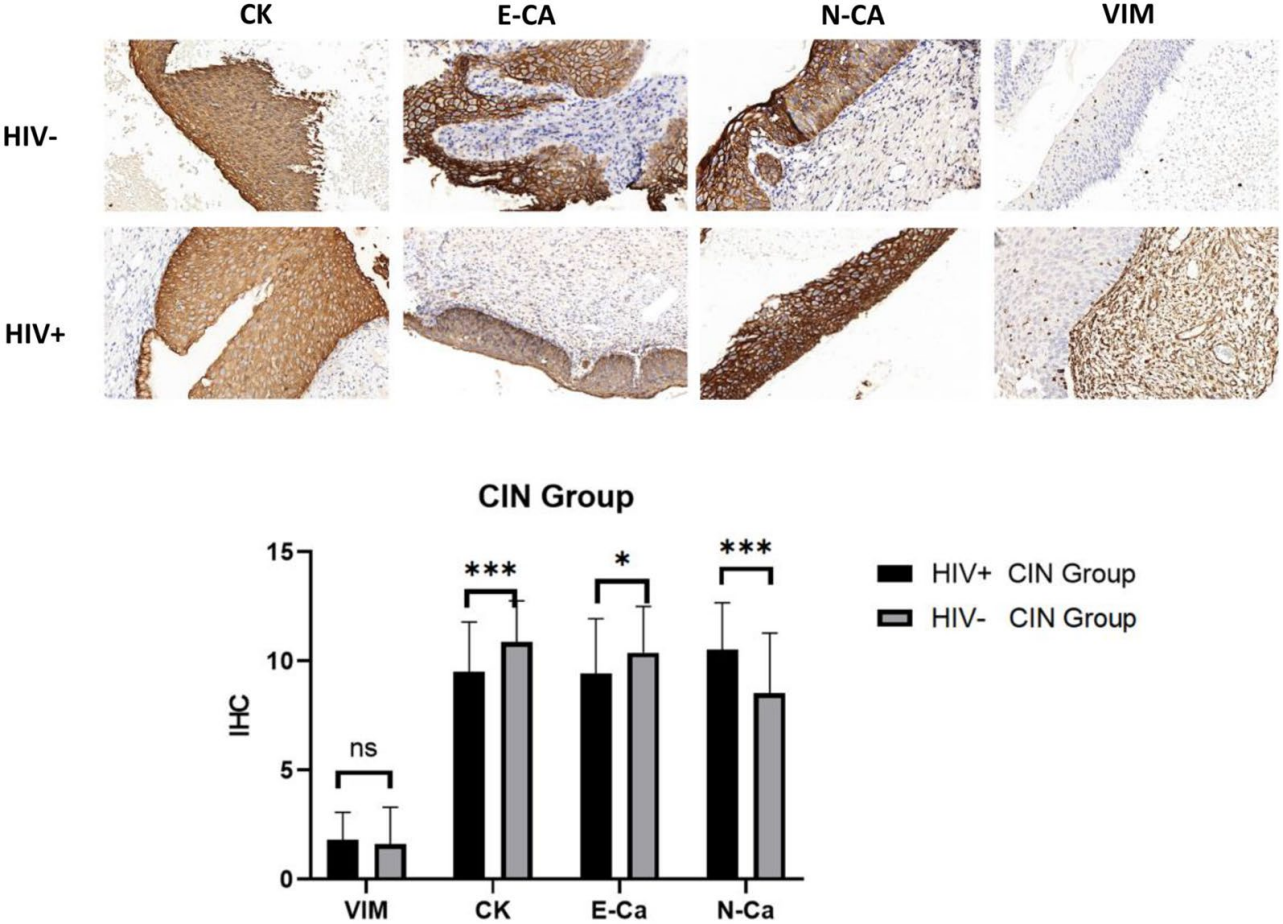
Immunohistochemistry was employed to compare the expression levels of EMT-related proteins in surgically resected cervical tissues from both HIV-positive and HIV-negative CIN patients. *CK* cytokeratin, *E-CA* E-Cadherin, *N-CA* N-Cadherin, *VIM* vimentin, *ns* non-significant; *P < 0.05; **P < 0.01; ***P < 0.001

To investigate whether HIV infection can induce EMT in cervical tissue, the expression levels of EMT-related protein markers were compared between HIV-positive and HIV-negative patients with different grades of cervical lesions. Immunohistochemical analysis was performed on surgical specimens from CIN patients with HIV infection (n = 54) and CIN patients without HIV infection (n = 64). There was no significant difference in age, CIN level, and lymphocyte count between the two groups (Table 1, all P > 0.05). Immunohistochemical analysis showed that, during the CIN stage, HIV-positive patients exhibited significantly decreased expression levels of E-cadherin and cytokeratin, along with increased expression levels of N-cadherin in cervical lesion tissues compared to HIV-negative patients (all P < 0.05, Fig. 1).

**Table 1:** Demographic and clinical characteristics of patients with different grades of cervical lesions

		Age (years)	CIN stage		Lymphocyte count (1 × 10^9^ cells/l)
CIN (I–III stages)	HIV- (n = 64)	36.74 ± 10.03	I	22	1.938 ± 0.533
			II	28	
			II	14	
	HIV+ (n = 54)	40.21 ± 11.75	I	19	1.717 ± 0.531
			II	20	
			III	15	
	Statistics	1.591	0.745		1.667
	P	0.115	0.689		0.100

		Age (years)	Cervical cancer stages		Lymphocyte count (1 × 10^9^ cells/l)
Cervical cancer (I–II stages)	HIV− (n = 22)	44.73 ± 10.02	I	16	1.839 ± 0.542
			II	6	
	HIV+ (n = 26)	44.05 ± 9.41	I	15	1.742 ± 0.617
			II	11	
	Statistics	− 0.213	1.178		− 1.498
	p	0.833	0278		0.135

*HIV* human immunodeficiency virus, *CIN* cervical intraepithelial neoplasia

**Fig. 2:**
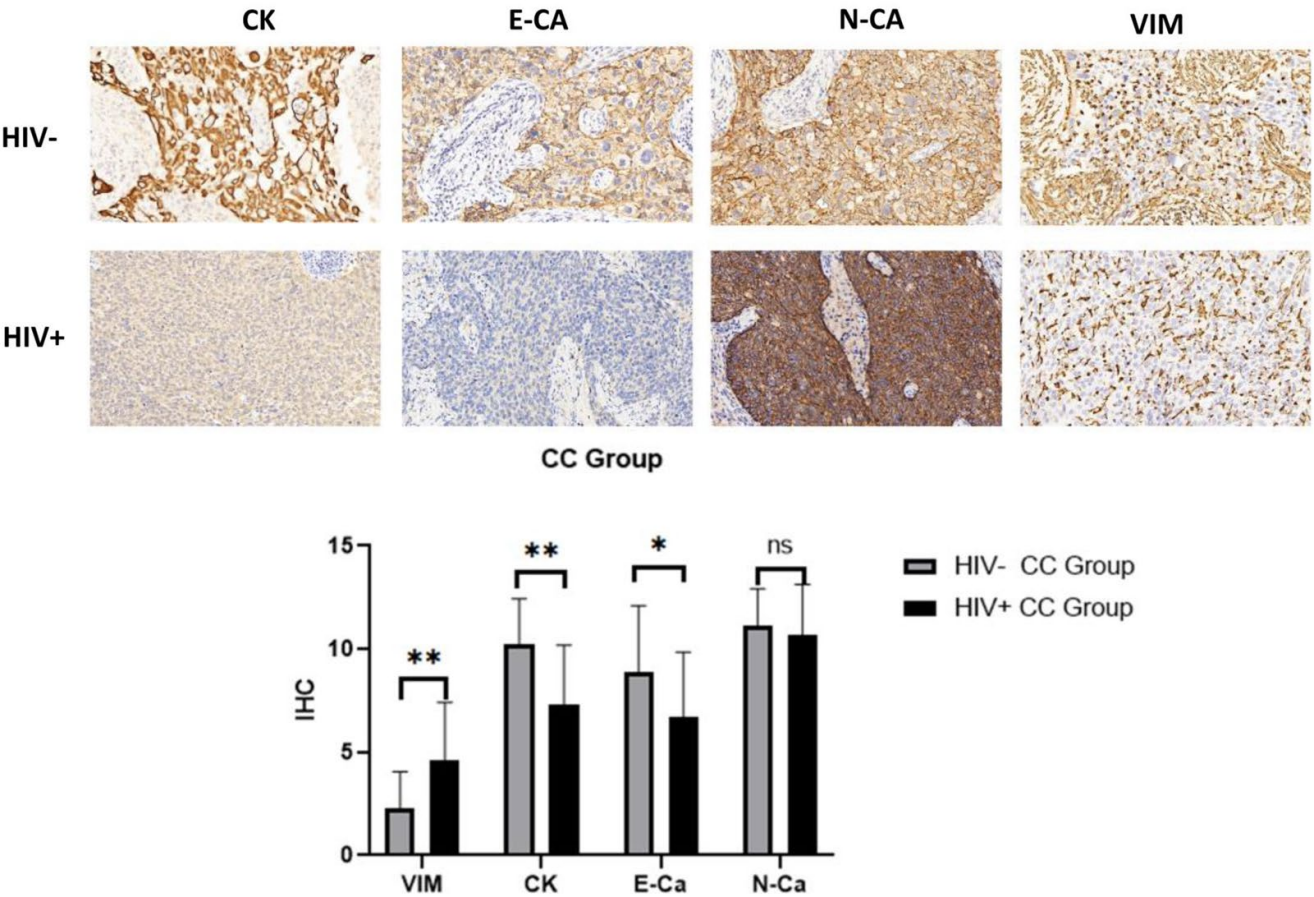
Immunohistochemistry was utilized to compare the expression levels of EMT-related proteins in surgically resected cervical tissues from both HIV-positive and HIV-negative patients diagnosed with cervical cancer at stages I and II. *CK* cytokeratin, *E-CA* E-Cadherin, *N-CA* N-Cadherin, *VIM* vimentin, *ns* non-significant; *P < 0.05; **P < 0.01; ***P < 0.001

The expression levels of EMT-related protein markers were also compared between the HIV-infected cervical cancer patients (n = 26) and HIV-negative cervical cancer patients (n = 22). There was no significant difference in age, cervical cancer stage, and lymphocyte count between the two groups (Table 1, all P > 0.05). Immunohistochemical analysis showed that during the cervical cancer stage, HIV-positive patients exhibited significantly decreased levels of E-cadherin and cytokeratin, and increased levels of vimentin in cervical lesion tissues as compared to HIV-negative patients (all P < 0.05, Fig. 2).

### HIV Tat and gp120 proteins induced EMT in SiHa cervical cancer cells

**Fig. 3:**
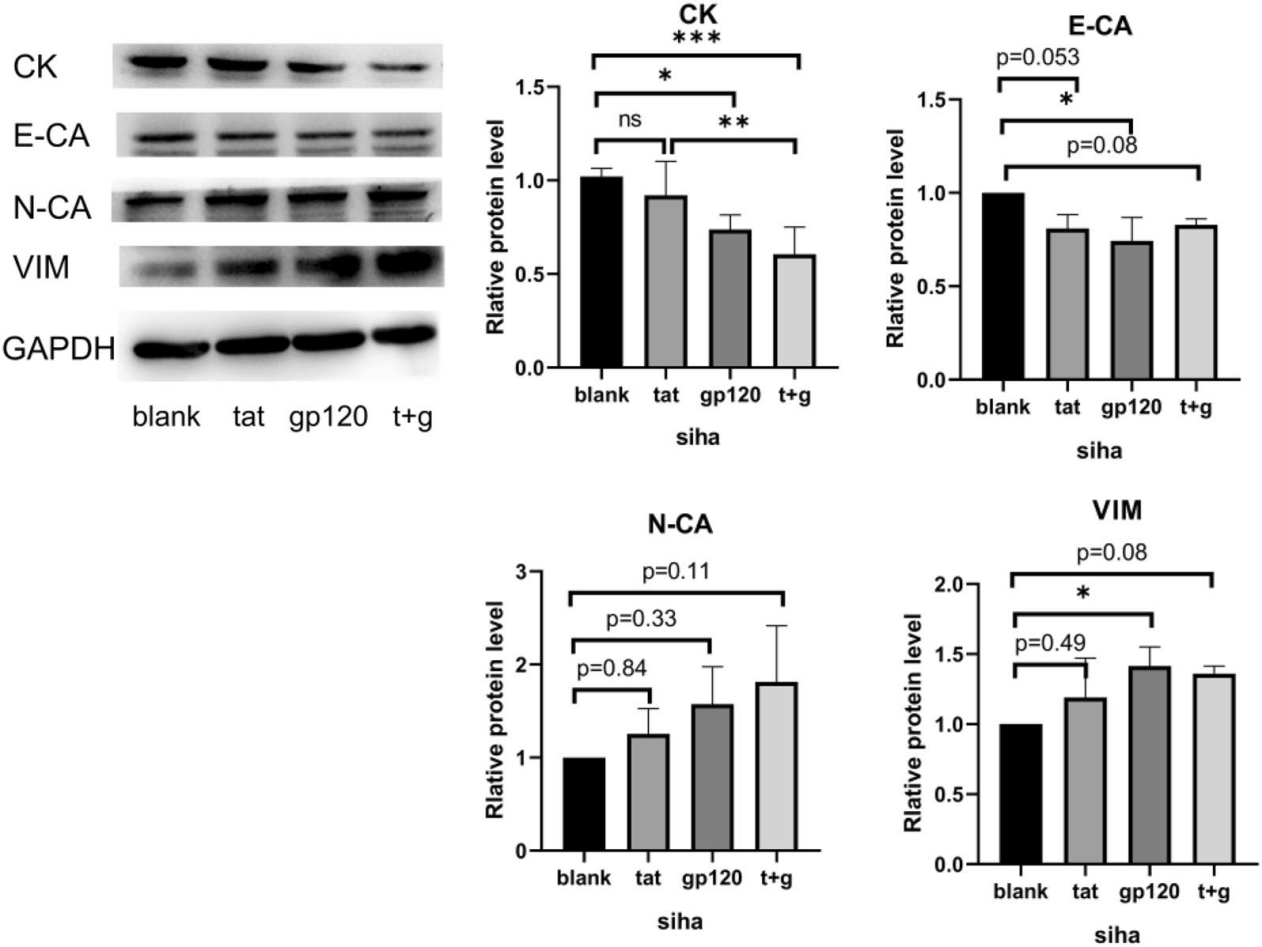
The expression levels of EMT-related proteins in SiHa cells following treatment with HIV-tat, gp120, or both. SiHa cells were seeded at a density of 1 × 10^5^ per well and exposed to HIV-tat and/or gp120 proteins, both at a concentration of 100 ng/ml, for 5 days. The culture medium containing HIV-tat and/or gp120 proteins was replaced daily during this period. After the treatment, Western blot was conducted to evaluate the expression of CK, E-cadherin, cytokeratin, vimentin, and N-cadherin. The experimental groups included a blank control group, an HIV-tat protein treatment group (tat), an HIV-gp120 protein treatment group (gp120), and a group treated with both HIV gp120 and tat proteins (t + g). *CK* cytokeratin, *E-CA* E-Cadherin, *N-CA* N-Cadherin, *VIM* vimentin, *ns* non-significant; *P < 0.05; **P < 0.01; ***P < 0.001

HIV gp120 and Tat proteins have been shown to induce the EMT phenotype in cervical epithelial cells [13]. To further confirm the role of HIV infection in inducing EMT in cervical tissue, human cervical cancer cells, SiHa were treated with HIV Tat and gp120 proteins (both at a dose of 100 ng/ml for 5 days), and the expression levels of EMT-related proteins were examined by Western blot. The results demonstrated that after treatment with HIV-Tat and gp120 proteins, SiHa cells exhibited upregulation of N-cadherin and vimentin expression levels, while E-cadherin and cytokeratin expression levels were downregulated (all P < 0.05, Fig. 3).

### HIV Tat and gp120 proteins enhanced the migration and invasion abilities of SiHa cells

**Fig. 4:**
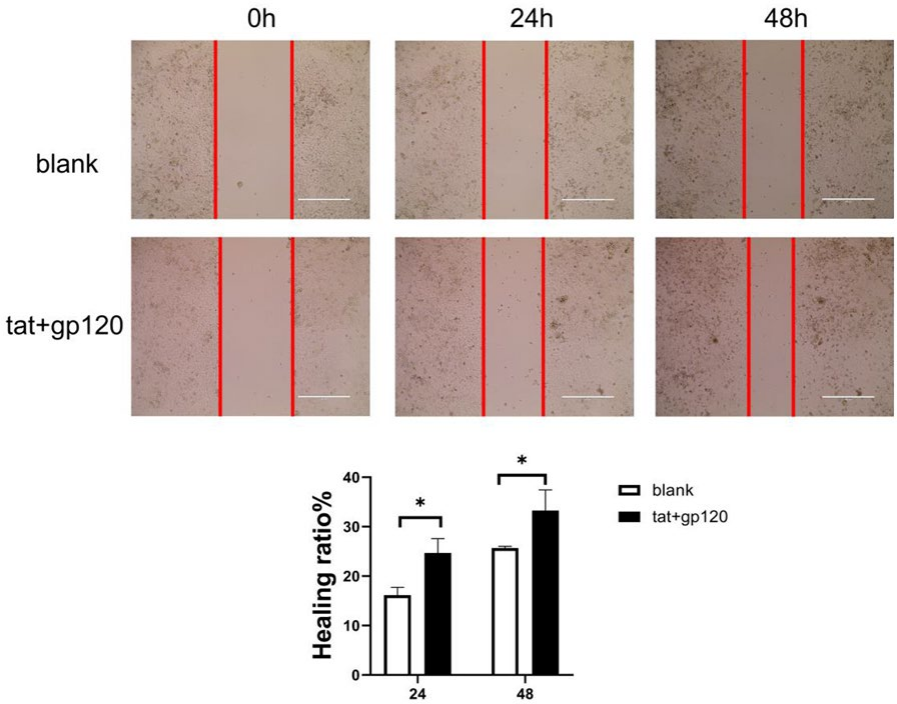
Changes in the wound healing rate of SiHa cells with/without HIV-tat and gp120 proteins treatment. SiHa cells were seeded in 6-well plates at a density of 1 × 10^5^ cells per well. In the treatment group, cells were exposed to 100 ng/ml of HIV gp120 protein and 100 ng/ml of Tat protein. The culture medium, along with HIV-tat and/or gp120 proteins, was replaced daily. When cell confluence reached 90%, a uniform scratch was made using a 200 µl pipette tip to create a wound. Subsequently, images of the wound area were captured at 0 h, 24 h, and 48 h using a microscope. Image J software was employed to calculate the wound area. *P < 0.05. Representative images from three repeated experiments are presented. The scale bar is set at 400 μm

Considering that HIV Tat and gp120 proteins have the potential to induce EMT in SiHa cells, their impact on the migratory and invasive capacities of cervical cancer cells was further investigated. Wound healing assay revealed that after treatment with HIV Tat and gp120 proteins (both at a dose of 100 ng/ml for 5 days), the wound closure of SiHa cells was enhanced at 24 (24.69 ± 2.89% vs.16.16 ± 1.55%, p=0.011) hours and 48 h (33.27 ± 4.19% vs. 25.68 ± 0.32%, p = 0.035) as compared with the untreated control group (Fig. 4).

**Fig. 5:**
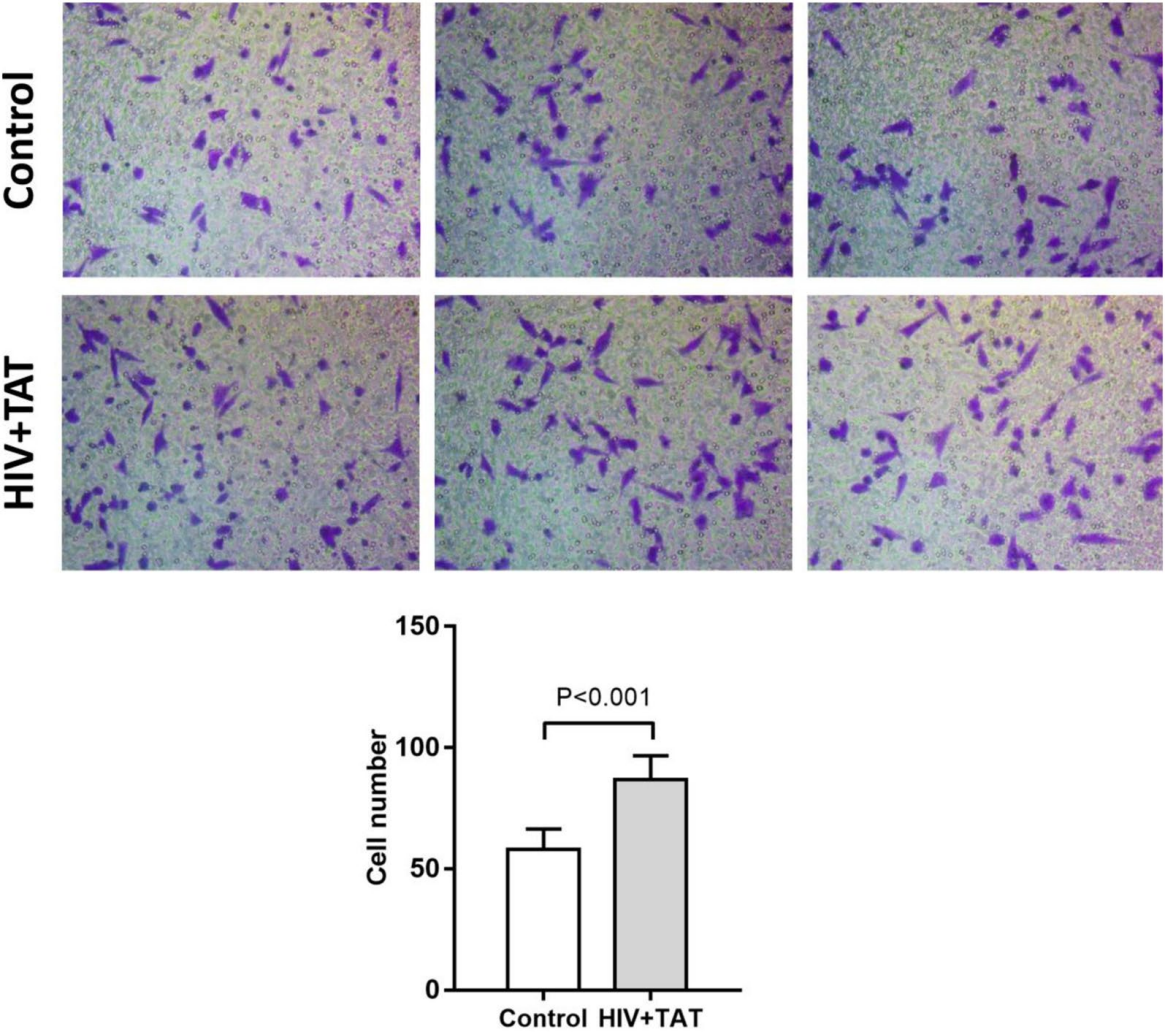
The transwell assay was employed to assess the effect of HIV gp120 protein and Tat protein on the invasiveness of SiHa cells. In the HIV gp120 + Tat protein treatment group, cells were exposed to HIV gp120 protein and Tat protein, both at a concentration of 100 ng/ml, for 5 days before being collected for the transwell assay. The transwell assay was conducted using a six-well transwell culture plate, with 1 × 10^5^ cells seeded in 1 ml of cell culture medium per well. The figure displays representative images from three independent experiments. The accompanying bar chart depicts the number of cells that transmigrated through the membrane in SiHa cells with or without HIV-tat and gp120 protein treatment

The invasion ability was assessed using the Transwell migration assay. The results showed that following treatment with HIV Tat and gp120 proteins, the invasion ability of SiHa cells significantly increased as compared to the blank control group (86.78 ± 9.92 vs. 57.89 ± 8.57 cells, p < 0.001, Fig. 5).

### Transcriptomic analysis of HIV Tat and gp120 proteins-treated SiHa cells

**Fig. 6:**
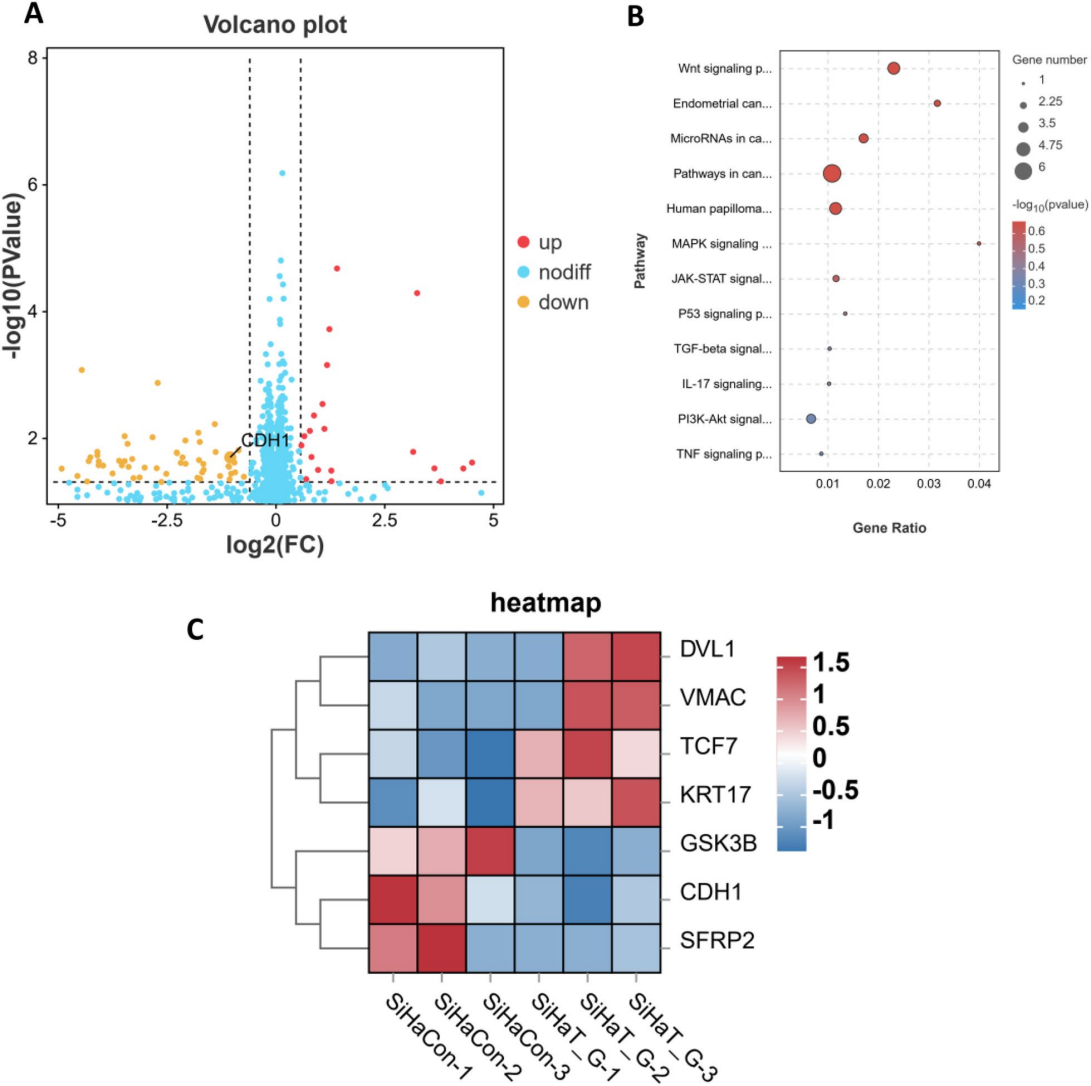
Transcriptomic analysis reveals significant mRNA expression changes in SiHa cells following HIV Tat and gp120 protein treatment. **A** Volcano plot displaying the differential expression of mRNAs. The vertical line represents a 1.5-fold change in expression (either upregulation or downregulation), while the horizontal line indicates the threshold for a 5% FDR. Red dots denote upregulated mRNAs, and yellow dots represent downregulated mRNAs. **B** Top 12 pathways associated with tumor biology functions, enriched with the differentially expressed mRNAs based on KEGG pathway analysis. The size of the bubbles indicates the number of enriched mRNAs, and the color reflects the p-value. The red frame highlights the Wnt signaling pathway, which is potentially linked to EMT. **C** Heatmap illustrating the expression pattern of key genes involved in the Wnt β-catenin pathway

To identify differentially expressed genes associated with EMT, transcriptome sequencing and bioinformatics analyses were performed. RNA sequencing revealed significant alterations in gene expression profile between the HIV Tat and gp120 protein-treated group and the untreated control group. Specifically, 22 genes were upregulated, and 77 genes were downregulated in the HIV Tat and gp120-treated group compared to the control group (Fig. 6A). Notably, the expression of the E-cadherin gene, CDH1, was significantly downregulated.

Further Gene Ontology (GO) and KEGG pathway enrichment analyses of the differentially expressed genes unveiled the top 12 tumor biology-related pathways (Fig. 6B), among which the Wnt signaling pathway is known to play a significant role in the regulation of EMT in cervical cancer SiHa cell carcinoma [14].

The gene expression profile of upstream genes of the Wnt/β-catenin signaling pathway was further analyzed through a heatmap. The analysis revealed upregulation of Dishevelled Segment Polarity Protein 1 (DVL1), Transcription factor 7 (TCF7), Keratin 17 (KRT17), and vimentin gene (VMAC) and downregulation of Glycogen synthase kinase 3β (GSK3β), secreted Frizzled-related protein 2 gene (SFRP2), and E-cadherin gene (CDH1) in the HIV Tat and gp120-treated group compared to the untreated control group (Fig. 6C). These findings suggested that HIV Tat and gp120 proteins treatment induced the activation of the Wnt signaling pathway as well as the upregulation of VMAC (p = 0.12) and the downregulation of CDH1 (p < 0.05) in SiHa cells.

### HIVgp120 and Tat proteins activate Wnt/β-catenin signaling in SiHa cells

**Fig. 7:**
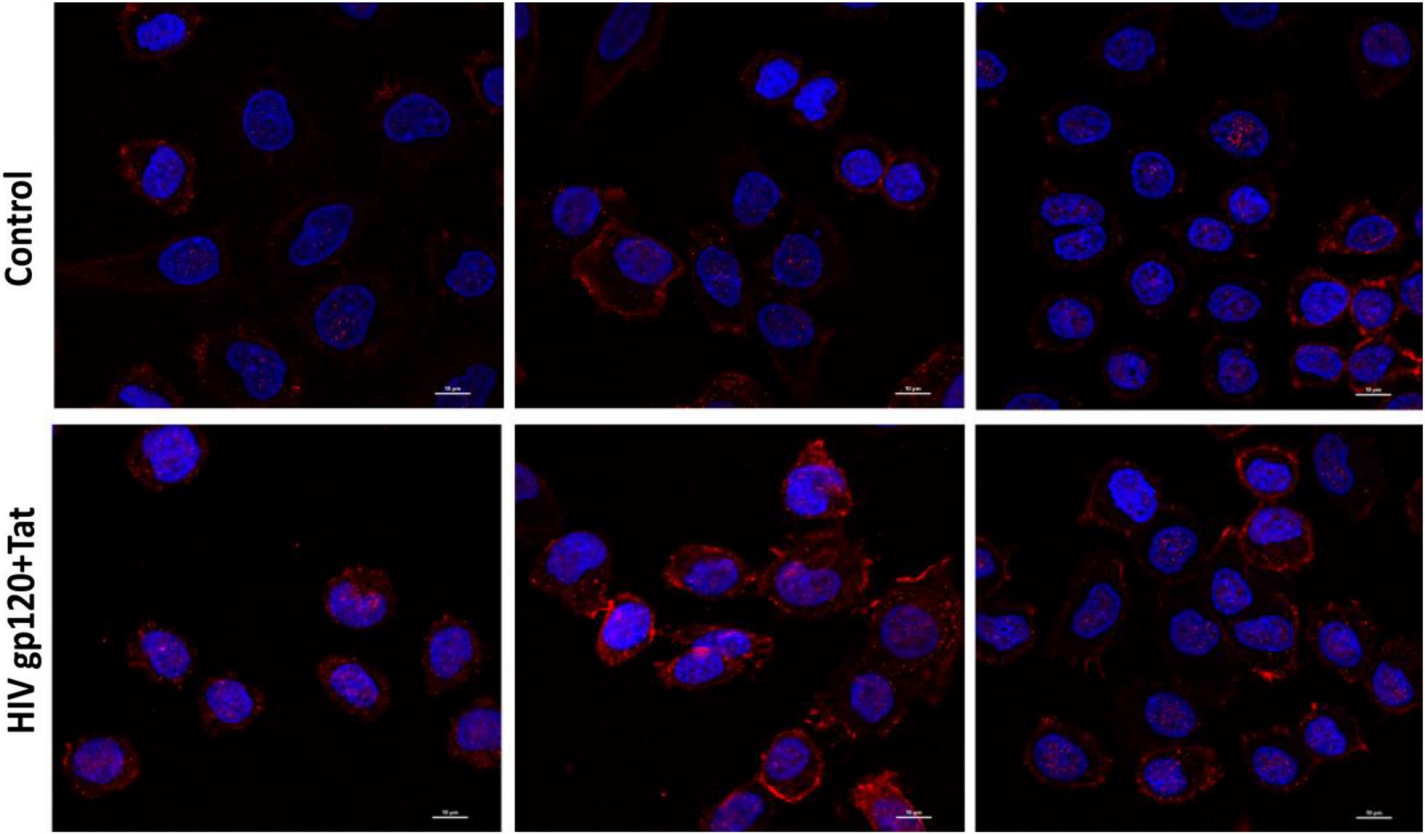
Immunofluorescence depicting the differential expression of β-catenin in SiHa cells following treatment with HIV-tat and gp120 proteins. Nuclei stained with DAPI appeared blue under ultraviolet excitation, while red fluorescein-labeled signals indicated positive β-catenin expression. The findings revealed cytoplasmic and nuclear expression of β-catenin in SiHa cells. Upon treatment with HIVgp120 and tat protein, an increase in β-catenin expression within SiHa cells was observed, including its accumulation in the nucleus

To further confirm the effect of HIVgp120 and Tat proteins on Wnt/β-catenin signaling pathway, immunofluorescence assay was performed. The results demonstrated that β-catenin was expressed in the cytoplasm and nucleus of SiHa cells. Following treatment with HIVgp120 and Tat proteins, the expression of β-catenin in SiHa cells increased; accompanied by nuclear accumulation (Fig. 7), further suggesting activation of the Wnt/β-catenin pathway.

## Discussion

In this study, we did not observe a significant difference in age between HIV-positive cervical cancer patients and HIV-negative cervical cancer patients (44.05 ± 9.41 vs. 44.73 ± 10.0 years, P = 0.833), contrary to the findings of previous reports [5, 6]. These inconsistencies may be attributed to the small sample size in our study.

EMT plays a crucial role in the processes of tumor initiation, invasion, and metastasis [15]. During EMT, cells undergo a loss of their original cellular polarity and intercellular adhesion, accompanied by the downregulation of epithelial markers including E-cadherin and cytokeratins, and the upregulation of mesenchymal markers such as N-cadherin and Vimentin [15]. In this study, immunohistochemical analysis revealed a significant decrease in epithelial markers (E-cadherin, cytokeratins) and a more pronounced increase in mesenchymal markers (N-cadherin, Vimentin) in HIV-positive patients compared to HIV-negative patients. These findings suggest an increased level of EMT in the cervical cancer tissues of HIV-positive patients, indicating that HIV infection may facilitate the progression of cervical lesions through EMT.

The HIV gp120 protein plays a crucial role in viral invasion and immune evasion processes as a component of the envelope glycoprotein complex, along with the transmembrane glycoprotein gp41 [15]. The HIV Tat protein plays a vital regulatory role by primarily activating the initiation and elongation of viral genome transcription within infected cells, thus initiating viral replication [16]. In addition, Tat protein can be secreted by HIV-infected cells into the extracellular environment or released from apoptotic and dying infected cells [17]. It has been reported that in HIV patients, prolonged exposure of the oral mucosal epithelium to free HIV particles and various sources of proteins can lead to the disruption of tight junctions within the oral mucosal epithelium, ultimately resulting in impaired mucosal epithelial function [18]. The interaction between HIV proteins gp120 and Tat with the mucosal epithelium disrupts tight and adherens junctions of oral epithelial cells, leading to reduced expression of E-cadherin and cytokeratin, activation of mesenchymal markers such as vimentin and N-cadherin, ultimately resulting in the occurrence of EMT [13].

In this study, the treatment of SiHa cells with Tat and gp120 proteins resulted in a significant decrease in epithelial markers (E-cadherin, cytokeratin) and a substantial increase in mesenchymal markers (N-cadherin, Vimentin). Additionally, Transwell and wound healing assays demonstrated an augmented migratory and invasive phenotype in SiHa cells following the exposure to HIV Tat and gp120 proteins. These findings suggest that HIV Tat and gp120 proteins play a role in promoting the progression of EMT in cervical cancer epithelial tissues.

The Wnt/β-catenin signaling pathway is crucial in regulating various developmental processes such as embryogenesis, organogenesis, and tissue homeostasis [19]. The hyperactivation of the Wnt/β-catenin signaling pathway has been linked to the initiation, progression, recurrence, and drug resistance of different types of cancers, including gynecological malignancies. [20]. The Wnt/β-catenin signaling pathway involves components such as Wnt factors, Dvl, KRT17, GSK-3β, Axin, SFRP2, β-catenin, T-cell factor/lymphoid enhancer factor (TCF/LEF) transcription factors, and APC [20]. The Wnt/β-catenin pathway signaling is primarily mediated by the TCF/LEF transcription factor family, leading to the activation of the Wnt/β-catenin pathway [21]. After activation of the Wnt/β-catenin pathway, β-catenin translocates to the cell nucleus, thereby promoting the expression of target genes to regulate cellular functions [22].

KRT17, a member of the intermediate filament family, has been shown to regulate the proliferation and migration of tumor cells. Overexpression of KRT17 has been associated with increased activity of β-catenin and elevated levels of Wnt target genes such as cyclin D1, c-Myc, and MMP7. This upregulation of KRT17 ultimately leads to increased expression of Vimentin, MMP-9, and Snail, while downregulating the expression of E-cadherin, promoting the occurrence of EMT [23, 24].

GSK3β has been implicated in triggering proteasomal degradation by promoting the phosphorylation of Snail and β-catenin, thereby regulating the transcription of E-cadherin. Inhibition of GSK3β expression leads to the stabilization of Snail and β-catenin, resulting in the downregulation of E-cadherin and promotion of EMT [25]. Simultaneously, upon activation of the Wnt/β-catenin pathway, Wnt protein induces the transduction of the cytoplasmic Dvl protein. Dvl, in turn, negatively regulates the activity of GSK-3β, leading to the activation of TCF-dependent transcription and promoting EMT [20].

The SFRP2 is a glycoprotein gene containing a Frizzled-like cysteine-rich domain, which allows it to bind to Wnt ligands or Frizzled receptors [26]. Knockdown of SFRP2 expression has been shown to activate the Wnt/β-catenin signaling pathway in cell lines, while overexpression of SFRP2 can inhibit Wnt/β-catenin activation and its downstream effects [27]. Activation of the Wnt/β-catenin signaling pathway has been shown to promote VMAC expression and up-regulate the level of Vimentin [28] while inhibiting the expression of the E-cadherin gene (CDH1) [29].

In this study, transcriptome sequencing revealed an expression pattern of EMT-associated proteins, indicating that HIV Tat and gp120 treatment induced EMT progression in cervical cancer SiHa cells. Enrichment analysis of differentially expressed genes identified the involvement of the Wnt/β-catenin pathway in EMT. Sequencing analysis of genes related to the Wnt/β-catenin pathway showed that compared to the control group, the HIV Tat and gp120 protein-treated group exhibited upregulation of TCF, KRT17, and DVL expression, downregulation of GSK3β and SFRP2 expression, upregulation of VMAC expression, and downregulation of CDH1 expression. Moreover, the immunofluorescence assay demonstrated that HIVgp120 and Tat proteins treatment induced elevated β-catenin expression with nuclear accumulation in SiHa cells. These findings suggest that HIV Tat and gp120 proteins can activate the Wnt/β-catenin pathway in SiHa cells, and the Wnt/β-catenin pathway may be one of the important pathways promoting EMT progression in cervical lesion tissues of HIV-infected patients. To the best of our knowledge, this is the first study reporting the effect of HIV Tat and gp120 proteins on EMT in cervical cancer.

There are still some limitations of the current study. First, the study primarily relied on in vitro experiments using SiHa cells to investigate the effects of HIV Tat and gp120 proteins on EMT. While this approach provides valuable insights into the cellular mechanisms, it does not fully capture the complexity of the in vivo microenvironment and interactions between different cell types. Therefore, these in vitro findings should be further validated in in vivo models or clinical studies. In addition, given the limited sample size in this study, a comparative analysis between the cervical cancer and CIN groups was not undertaken. Also, we did not investigate the association of expression results with various stages of CIN/cervical cancer due to the relatively small sample size of this study. Larger sample sizes and diverse populations should be considered in future studies to enhance the robustness and applicability of the findings.

## Conclusions

Treating SiHa cells with HIV Tat and gp120 proteins induces EMT and activates the Wnt/β-catenin pathway, suggesting that the Wnt/β-catenin pathway may play a crucial role in promoting EMT progression in cervical lesion tissues of HIV-infected patients. These findings provide insights into the pathogenesis of HIV-associated cervical cancer and highlight the importance of optimizing prevention and treatment strategies for HIV-positive women with cervical cancer.

## Data Availability

The datasets used and/or analysed during the current study are available from the corresponding author on reasonable request.

## Abbreviations

AIDS: Acquired immunodeficiency syndrome
HIV: Human immunodeficiency virus
HPV: Human papillomavirus
EMT: Epithelial–mesenchymal transition
CIN: Cervical intraepithelial neoplasia
LSD: Least significant difference
ANOVA: One-way analyses of variance
GO: Gene ontology
DVL1: Dishevelled Segment Polarity Protein 1
TCF7: Transcription factor 7
KRT17: Keratin 17
VMAC: Vimentin gene
GSK3β: Glycogen synthase kinase 3β
SFRP2: Secreted frizzled-related protein 2 gene
CDH1: E-cadherin gene
TCF/LEF: T-cell factor/lymphoid enhancer factor
CDH1: E-cadherin gene
